# The Incidence of Dental Caries in Children with Down Syndrome: A Systematic Review and Meta-Analysis

**DOI:** 10.3390/dj10110205

**Published:** 2022-10-31

**Authors:** Mariana Martins, Paulo Mascarenhas, José Grillo Evangelista, Isabel Barahona, Vitor Tavares

**Affiliations:** 1 Instituto Universitário Egas Moniz, Quinta da Granja, Monte de Caparica, 2829-511 Almada, Portugal; 2 Centro de Investigação Interdisciplinar Egas Moniz (CiiEM), Instituto Universitário Egas Moniz, Quinta da Granja, Monte de Caparica, 2829-511 Almada, Portugal

**Keywords:** Down syndrome, children, adolescents, caries

## Abstract

Scientific evidence regarding the incidence of dental caries in Down syndrome (DS) patients is limited and sometimes presents divergent opinions among authors, making it difficult to reach definitive conclusions. We aimed to evaluate the caries incidence in the DS pediatric population and compare it against healthy controls. The search was performed using 4 universal databases: Cochrane, B-on, Biomed, and PubMed. The selected articles were synthesized and subsequently evaluated according to an adaptation of the Quality Assessment Checklist for Prevalence Studies risk of bias tool, and analysis charts were performed by the Risk of Bias visualization tool (ROBVIS). Statistics and graphs were performed by Open Meta Analyst and JASP software. The confounding effect on caries incidence of the following factors was evaluated through meta-regression: age, Male/Female (M/F) ratio, DMFT, dmft, and study geographic location. Overall, the incidence of caries in the DS population was 49.9%, whereas in the control population was 63.4%. The M/F ratio, DMFT, and dmft significantly affected the incidence of DS individuals (*p*-value < 0.05). The evidence regarding the lower pooled incidence of caries in individuals with DS regarding controls is limited by the few scientific reports available and cross-section designs. Therefore, further studies are needed to confirm these results.

## 1. Introduction

Down syndrome (DS) is the most common autosomal abnormality in children of mothers aged over 30 years [1]. In humans, it is normal for each cell to have 46 chromosomes. However, in DS, in all or some cells, there is an extra copy of chromosome 21, which is responsible for its physical and developmental characteristics. The prevalence varies from 1 in 800–1200 live births and the carriers of this syndrome are usually identified at birth, with a distinct physical appearance, low growth and developmental delay [2].

At the oral level, patients with DS have underdeveloped the middle third of the face. In association with other characteristics, this feature causes palatal atresia and a narrowing of “V” shaped palate with a high arch, resulting in severe repercussions in the stomatognathic system. The maxillary sinus is hypoplastic in 90% of patients, and the bones of the face are smaller than those of normal individuals. Craniofacial dysplasia also frequently causes anterior open bite and pro-inclination of the lower incisors. Other oral anomalies also present in patients with DS are periodontal disease, chronic respiratory infections with repercussions in mouth breathing, and xerostomia. Enamel hypocalcification, fusion, twinning and decreased tooth root length are also present, which can cause mechanical difficulties [1,3,4,5]. Another common feature is periodontal disease in the form of invasive periodontitis, resulting from the fragile immunological system of patients with DS, that leads to difficulties fighting the microorganisms of the biofilm. However, there is a direct, or supporting action of this immunosuppression with dental caries. Here, dental cavitation results from enhanced colonization of microorganisms, causing demineralization of the tooth surface. After the mineral loss, the formation of a cavity is generally observed [6,7,8,9].

Due to their various health problems, patients with DS have to consume a variety of medication, which has glucose as a constituent, which is a factor for a higher incidence of caries in these patients [10].

Another problem that may be associated with the incidence of caries is the fact that these patients present a very precarious oral hygiene due to their muscular hypotonicity and due to their behavior during dental appointments. Furthermore, patients with DS may present behaviors of stubbornness, impulsiveness and non-cooperation, leading to the necessity of techniques that are not always available, such as systematic desensitization, tell-show-do, positive reinforcement, control by sedation with nitrous oxide, or perform treatments with general anesthesia [11].

However, despite the autosomal characteristics of patients with DS, some authors have reported a lower incidence of caries disease in patients with DS than in healthy controls, suggesting that characteristics such as diastema, delayed eruption, more alkaline saliva, dental agenesis and macroglossia are protective factors for dental caries [6,9,12].

Therefore, the objective of this systematic review and meta-analysis is, through the selective collection of scientific evidence, to globally assess the incidence of caries in the pediatric population with DS against the pediatric population without DS and to study the impact of confounding factors on this outcome.

## 2. Materials and Methods

This systematic review and meta-analysis followed the preferred reporting items for systematic reviews and meta-analyses guidelines (PRISMA), Table A1 [13].

The clinical question underlying this review was structured in the PECO form, with “*p*” being pediatric patients, “E” being exposed to the SD risk factor, “C” being compared with a healthy control group and “O” being the incidence of dental caries.

The present study followed other studies carried out by Deps et al. (2015), Robertson et al. (2019) and Silva et al. (2020) [14,15,16], in order to update these past revisions. It covers cross-sectional, i.e., observational epidemiological studies to answer the following questions: What is the incidence of dental caries in pediatric subjects with DS (up to 18 years of age), and in this population DS is a risk factor for caries?

### 2.1. Data Sources and Search Strategy

The authors carried out a systematic search on 4 universal digital databases: Cochrane, B-on, Biomed, and PubMed, using the following combinations of terms: (Children [Mesh] AND Down Syndrome [Mesh] AND Dental Cavities [Mesh]); (Adolescent [Mesh] AND Down Syndrome [Mesh] AND Dental Cavities [Mesh]); (Children [Mesh] AND Trisomy 21 [Mesh] AND Dental Cavities [Mesh]); (Adolescent [Mesh] AND Trisomy 21 [Mesh] AND Dental Cavities [Mesh]).

The search in the databases was initially carried out in December 2020 and updated in January 2021.

The systematic review and meta-analysis protocol was registered at PROSPERO register (CRD42020192321).

### 2.2. Inclusion Criteria

All studies needed to include pediatric patients with Down Syndrome up to 18 years of age, with primary, mixed or definitive dentition. Furthermore, studies were required to contain the associated incidence of caries, or data that would allow its calculation.

### 2.3. Selection Process

After databases screening, results were imported to Mendeley reference manager to remove replicates. Next, the articles found were analyzed and selected if they met inclusion criteria. The selection was carried out independently by M.M. and J.G. based on reading titles and abstracts and, later, validated by reading the full articles. If necessary, a third element (I.B.) would resolve disagreements.

### 2.4. Data Extraction

Data were extracted from the selected articles to obtain a synthesis table. Items collected were: authors and year of publication, methodology and type of assessment tool used, sample size both in the pediatric group with DS and in the control group, referring to gender and its ratio, type of dentition, caries incidence, decayed, missing and filled-in permanent teeth or primary teeth (DMFT/dmft), age, country and continent, and latitude of the place where the study was conducted.

### 2.5. Methodological Quality Assessment

The articles were subsequently evaluated according to an adaptation of the Quality Assessment Checklist for prevalence studies (Hoy et al., 2012) [17]. For each completed criterion, the study obtained a score ranging from 0 to 13. Articles scoring between 0–4 points were considered low risk, between 5–8 points moderate risk and between 9–13 points high risk. Studies with a score of up to 8 were accepted for meta-analyses.

The following subjects were assessed for risk of bias: if the study group was close to the national population, selection of study groups (confirmation of the diagnosis with DS through genetic tests or monitoring centers and confirmation of the presence of a healthy group); if the evaluations and data were taken in the same way (data obtained from the patient, evaluation of dental caries, DMFT/dmft indexes); control of factors that may interfere with the results obtained (gender and age); if there was a standard criterion for case and control; if the description of the subjects was well defined; and reliability and validity. Analysis charts were performed by the Risk of Bias visualization tool (ROBVIS).

### 2.6. Data Synthesis and Meta-Analysis

The incidence of caries for individuals with DS and controls, consisting of the ratio between individuals with caries and the total assessed, was extracted from each article selected for meta-analysis (MA). Subsequently, its global average incidence was calculated through MA.

The MA was performed with the values of the incidence transformed by the square root arcsine function, and later reconverted in the original units through the inverse function to comply with the statistical assumptions of the MA of proportions of counted data. This approach makes it possible to standardize incidences by mitigating any biases during MA. In the meta-regression and forest plot graphs, the meta-analytical values of the incidence of caries are shown reconverted in the original units.

The meta-analysis was adjusted using the method of DerSimonian & Laird (1986) [18], considering the presence of randomized effects. The degree of disagreement associated with the results was assessed using the I^2^ heterogeneity index, regarded as high whenever it exceeded 50%.

JASP software (JASP Team) built the funnel and radial plots to evaluate eventual publication bias. All other statistics and meta-analyses, including associated plots, were performed using the Open Meta Analyst software [19].

Meta-regressions were performed to assess possible causes of heterogeneity in the meta-analysis. In this sense, the effect of covariables such as age, male/female ratio (M/F), DMFT and dmft indices, and latitude was evaluated. The meta-regression of the variables was also assessed in a combined way whenever the statistical result of each isolated one was significant. The meta-regressions resulted in a coefficient for each covariate, which measured the effect of the variable (or variables) under study on the incidence of caries. They were considered statistically significant if they presented *p*-value < 0.05.

## 3. Results

**Figure 1:**
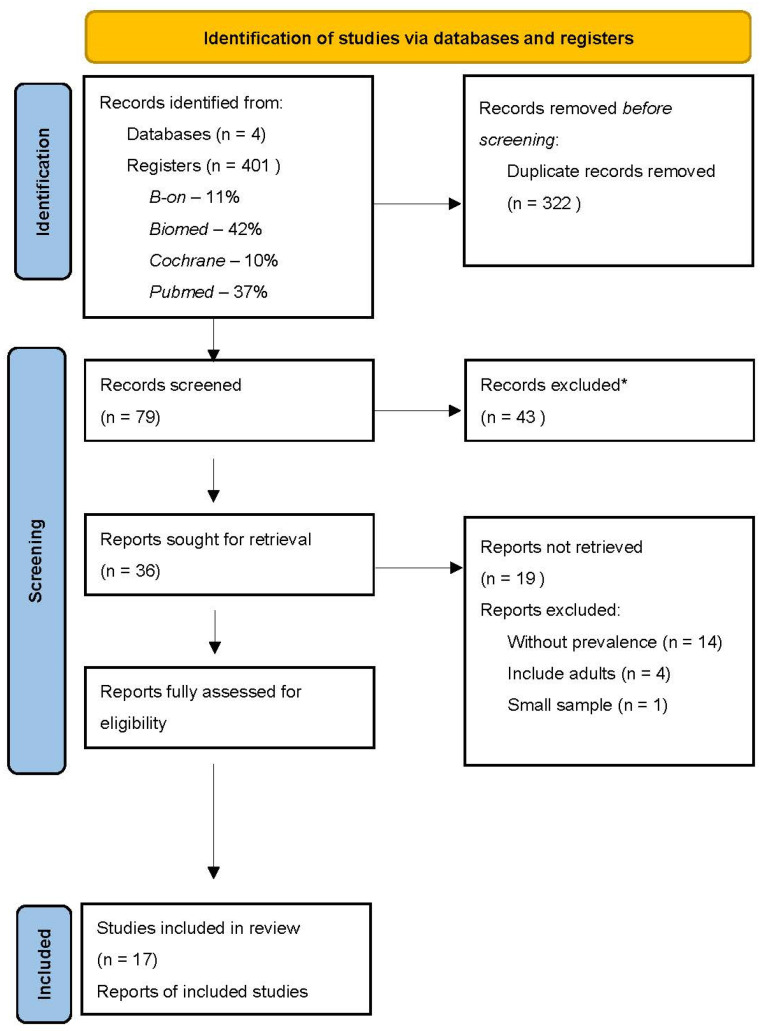
PRISMA flow chart illustrating the review search and screen process. n—number of records. * Articles excluded after reading the title and abstract do not correspond with the review of the theme subject.

Over 400 potentially relevant articles were identified in the electronic literature databases (Figure 1). Then, articles were imported to Mendeley to eliminate replicates, leaving 79 articles. After reading the title and abstract, more than 43 articles were excluded, leaving only 36 for a full reading. After thoroughly reading the 36 articles previously selected and following the inclusion criteria, 19 articles were eliminated, leaving 17 articles to use in this review. In this selection, there was 100% reliability among the reviewers.

Table 1 shows the characteristics of the studies. The studies were conducted in South American countries, Asia, Europe and Africa. The studies had different sample sizes for the DS and control groups; however, the study by Singh et al. [20] and Scalioni et al. [21] used the same sample size for both groups (DS patients and control patients), in order to obtain more reliable results. The age, M/F ratio, caries incidence and their DMFT/dmft indexes, and the countries in which the studies were carried out are also shown.

### 3.1. Risk of Bias

**Figure 2:**
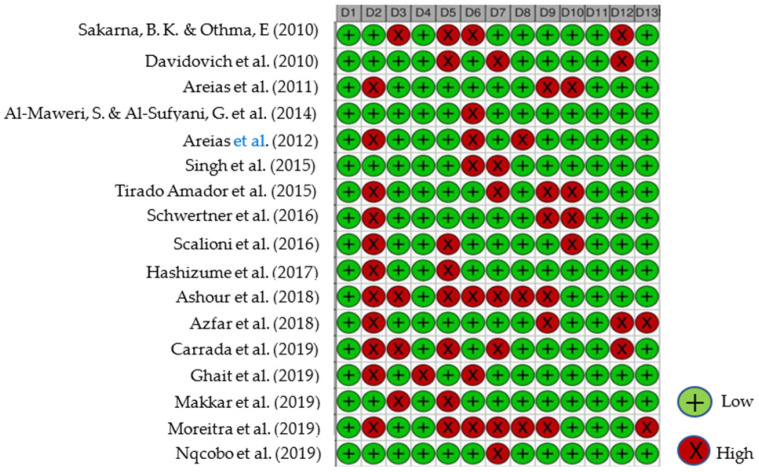
Studies Traffic light risk of bias plot. D1—Population representativeness; D2—Target population; D3—DS group; D4—Control group; D5—Data collection in the same way; D6—Data collection through the subject; D7—Default criteria; D8—Well described subject; D9—Reliability and validity; D10—Cavitation incidence; D11—DMFT/dmft; D12—Genre; D13—Age. Articles evaluated [20,21,22,23,24,25,26,27,28,29,30,31,32,33,34,35,36] (from top to bottom).

**Figure 3:**
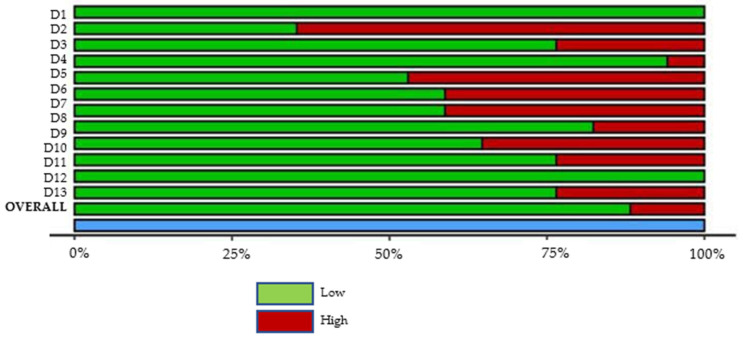
Studies Summary risk of bias plot. D1—Population representativeness; D2—Target population; D3—DS group; D4—Control group; D5—Data collection in the same way; D6—Data collection through the subject; D7—Default criteria; D8—Well described subject; D9—Reliability and validity; D10—Cavitation incidence; D11—DMFT/dmft; D12—Genre; D13—Age.

All reviewed articles were evaluated for risk of bias since all contained quantitative data and could potentially contribute to the meta-analysis. The analysis results graphs are displayed in Figure 2 and Figure 3. After a careful critical assessment of the risk of bias, all selected articles had a low/moderate risk of bias, thus allowing their entry into the meta-analysis study.

### 3.2. Meta-Analysis

**Figure 4:**
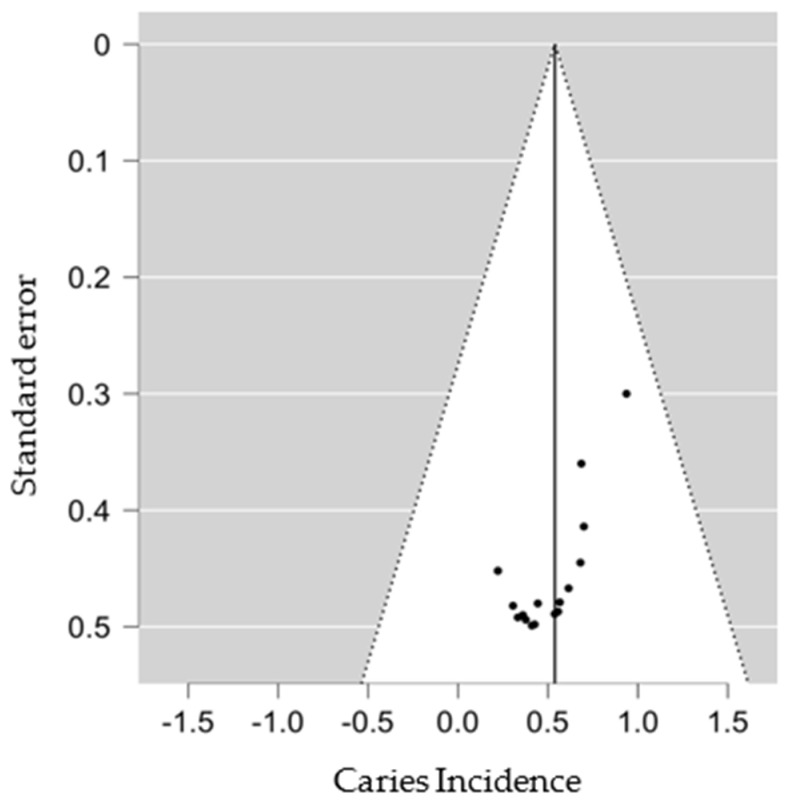
SD group Funnel Plot.

**Figure A1:**
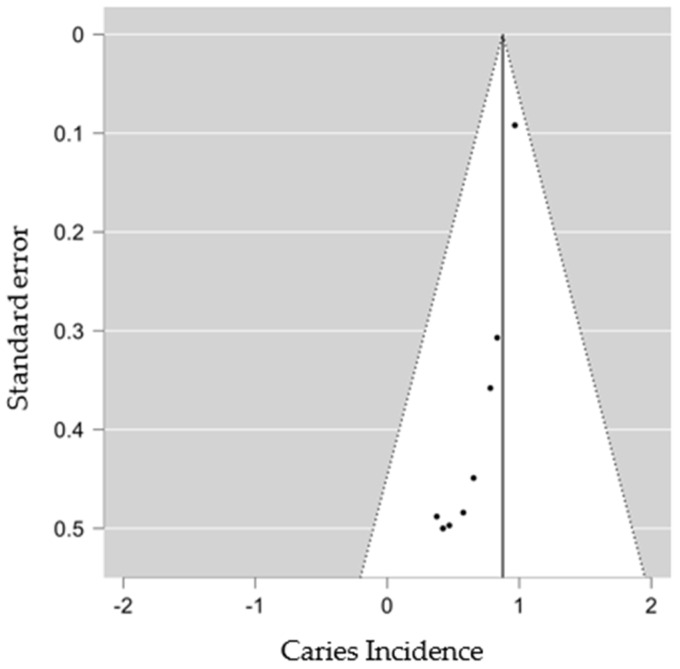
Control group funnel lot.

We used the funnel plot to assess for an eventual publication bias, and the results obtained did not suggest publication bias (Figure 4 and Figure A1).

**Figure 5:**
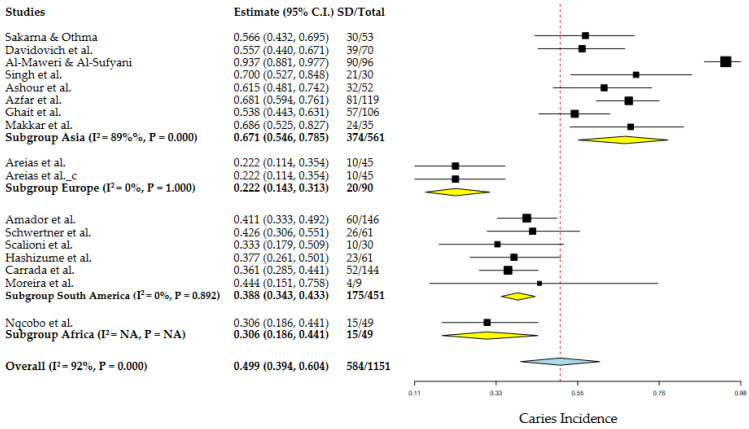
Geographic subgroup meta-analysis (Asia, Europe, South America and Africa) of caries incidence in DS patients. Articles references (from top to bottom): Subgroup Asia [20,22,23,25,30,31,33,34], Subgroup Europe [24,26] and Subgroup Africa [36].

**Figure A2:**
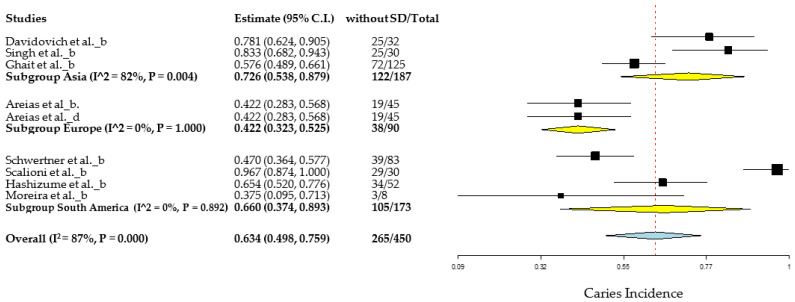
Geographic subgroup meta-analysis (Asia, Europe and South America) of caries incidence in control patients. Articles references (from top to bottom): Subgroup Asia [20,23,33], Subgroup Europe [24,26] and Subgroup America [21,28,29,35].

In the MA for the group with DS, 17 articles were used with a total of 1151 individuals, 584 individuals had dental caries, giving an average global incidence of 49.9%, with an associated heterogeneity of 92% (Figure 5). On the other hand, the control group included 9 articles, with a total of 450 individuals, 265 present dental caries giving an incidence of 63.4%, with an associated heterogeneity of 87%, in the same way, that in the pediatric group with DS the authors have had an incidence with high uncertainty (Figure A2). Potential covariates behind these heterogeneities were evaluated through subgroup MA and meta-regressions. In this context, the authors analyzed the role of geography in the incidence of caries, through sub-group MA for geographic regions and meta-regression for latitude. The role of other factors, such as age, M/F ratio, DMFT and dmft, was also assessed by meta-regressions. Among the results of the different geographic subgroups evaluated, the relationship between Asia, Europe, South America and Africa and the degree of heterogeneity associated between the continents was 92%, indicating that the average incidence of these continents was not similar. On the other hand, the results from Europe, South America and Africa did not show heterogeneity (I^2^ = 0%).

Regarding the control group, among the different subgroups, Asia, Europe and South America, there was a heterogeneity of 87%, thus indicating that the average incidence of caries between these continents is not similar (Figure A2).

### 3.3. Age

**Figure A3:**
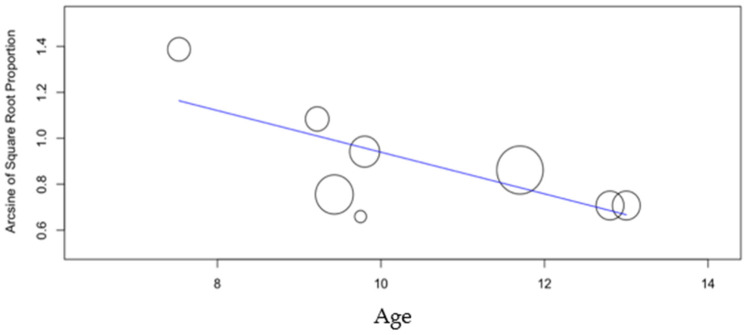
Meta-regression of age versus caries incidence for the control group.

The meta-regression of the mean age revealed no effect on the DS group caries incidence *(p*-value = 0.905) but a significant positive one (*p*-value < 0.001) in the control group, with increasing age associated with higher caries incidence (Figure A3).

### 3.4. Male/Female Ratio

**Figure 6:**
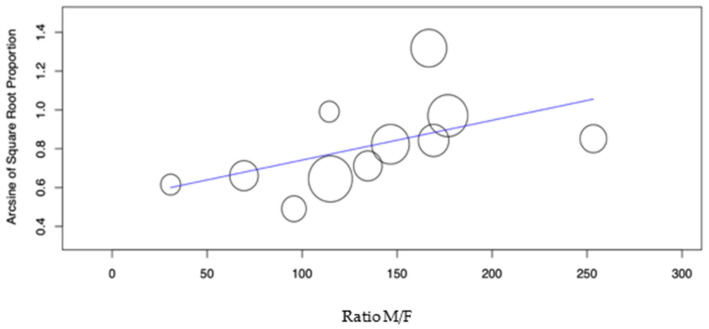
Meta-regression of the Ratio M/F variable versus caries incidence for the DS group.

The sample’s Male/Female (M/F) ratio showed a significant effect on the DS group caries incidence (Figure 6, *p*-value = 0.042). In the control group, the same factor showed a non-significant effect (*p*-value = 0.463)**.**

### 3.5. DMFT/dmft Indices

**Figure 7:**
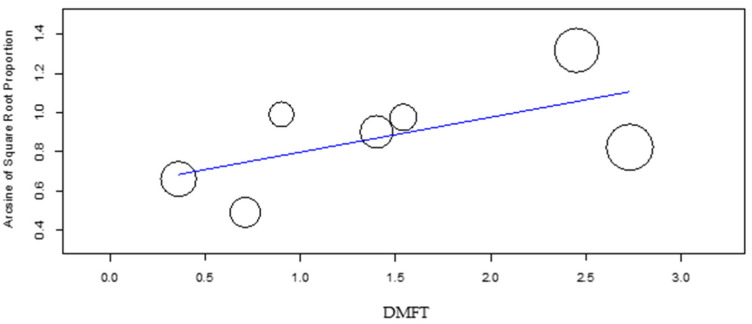
Meta- regression of DMFT versus caries incidence for the DS group.

**Figure 8:**
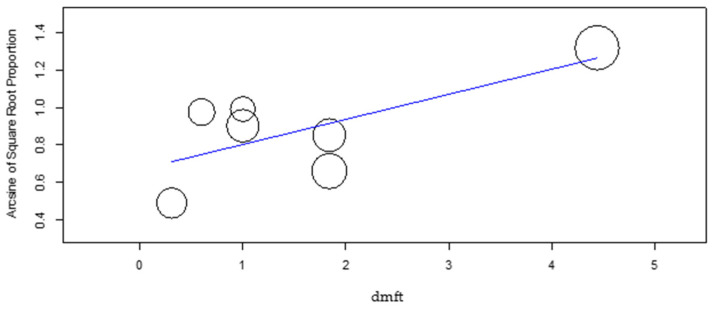
Meta-regression of dmft versus caries incidence for the DS group.

The meta-regression of the mean DMFT and dmft (Figure 7 and Figure 8) revealed a significant effect of these factors on the DS group caries incidence (*p*-value = 0.048 and *p*-value = 0.007, respectively), with increasing DMFT and dmft associated with higher caries incidence. In the control group, the DMFT and dmft have revealed no effect on caries incidence.

### 3.6. Latitude

The meta-regressions of the latitude revealed no effect on the DS and control group’s caries incidence (*p*-value = 0.214 and *p*-value = 0.652, respectively).

## 4. Discussion

The present study is a systematic review and MA that follows others performed by Deps et al. (2015), Robertson et al. (2019) and Silva et al. (2020) [14,15,16]. The studies used were cross-sectional, and the results evaluated caries incidence in pediatric patients with DS and controls.

Seven of the nine studies that evaluated the control group indicated that children and adolescents with DS had a lower incidence of caries when compared to controls. There are several possible reasons for this lower caries rate in children with DS. In their studies, Areias et al. [24] related the low incidence of caries to the fact that the teeth of patients with DS erupt later, therefore, subject to cariogenic factors for a shorter period. This study also associated these patients with bruxism, which smoothed the occlusal surfaces due to tooth friction, resulting in better self-cleaning and caries prevention [24]. Other factors that may explain the lower incidence of caries are changes in the salivary glands. Individuals with DS show changes in the proportion of electrolytes in saliva, increasing pH and bicarbonate levels that are associated with lower amounts of *Streptococcus mutans* (*S. mutans*) compared to the average amounts found in the general population [16,24,26,37,38].

In the study that Hashizume et al. [29] performed, reports that children with DS had higher IgA concentrations than children without the syndrome. The IgA protects against caries by inhibiting bacterial adhesion caused by the inhibition of enzymes and toxins, performing an active effect with other salivary components. This synergistic effect contributes to the control of the cariogenic microbiota [29].

Scalioni et al. [21] quantified by fluorescence in situ hybridization (FISH) cariogenic bacteria in the saliva of children and adolescents with DS compared to healthy control patients. They found that children and adolescents with DS had a lower density of *S. mutans* and a higher density of *Streptococcus sobrinus*, the latter being associated with the development of caries, especially on smooth surfaces [21,26].

In addition to all the aforementioned causes, dental morphological anomalies can also be considered, as they are common in patients with DS. The most frequent abnormalities are diastema, conoid teeth, microdontia and agenesis (which appear 10 times more in patients with DS than in the general population). These factors decrease susceptibility to caries, as this morphology allows for easy cleaning of all dental surfaces [1,5,24,39,40].

However, when assessing age in the DS pediatric group, the incidence decreased with increasing age. Shukla, D. et al., state that the increased incidence of caries may be associated with muscle weakness and inadequate muscle coordination, interfering with daily hygiene procedures [41]. Still, Scalioni, F. et al. [21] reports that children with DS and under 10 years old need more supervision by adults concerning oral hygiene since, in most cases, they are unable to brush their teeth independently [26]. However, with advancing age, when they reach adolescence or the age group of young adults, they want to become independent and brush their teeth on their own, which impacts oral hygiene [42].

This study evaluated the influence of the DMFT/DMF indexes, with only the DMFT index in the pediatric group, with DS being significant.

Castilho, A. & Marta, S. [43] evaluated caries incidence in patients with DS after insertion in a preventive program. The individuals examined had low caries rates (DMFT and dmft). After 12 months of follow-up, the caries incidence was evaluated using the same indices and there were only 4 new lesions in the DMFT index. However, the dmft values were lower at the end of 12 months. These results highlight the importance of introducing preventive programs to control dental caries better [43].

Oral hygiene and fluoride application are other prevention methods. It is recommendable to brush tooth twice a day with fluoride toothpaste. In order to facilitate brushing in children, parents should assist by placing toothpaste on a soft, age-appropriate sized toothbrush and help with execution and learning [44].

Fluoride in the caries process acts through cariostatic mechanisms, interfering in preventing enamel demineralization when there is supersaturation of the ion in oral fluids, partially inhibiting the metabolic activity of bacteria, not allowing the production of acids, especially lactic acid, thus favoring for remineralization [45].

Finally, it is important to change eating habits, prioritizing the consumption of vegetables, fruits and vegetables, reducing the amount and frequency of carbohydrates. It is also known that breast milk, together with other carbohydrates, is highly cariogenic [44].

It is relevant to mention that there were limitations in carrying out the review, as the included studies were mainly cross-sectional and had small, non-randomized samples.

## 5. Conclusions

Through the selective collection of scientific evidence, the incidence of caries in the pediatric population with DS versus the pediatric population without DS was globally evaluated and the impact of confounding factors on this outcome was studied.

However, based on the results of this study, it appears that, according to current scientific evidence, there is a lower incidence of caries in pediatric patients with DS than in healthy pediatric patients.

Thus, it is important to better plan how the study will proceed and carry out more high-quality clinical trials in the future, allowing studies with less heterogeneity and the possibility of evaluating caries in longer follow-ups, to confirm with greater precision the results obtained here, as well as the identification of clinic guidelines to reduce the incidence of caries in the long term.

## Figures and Tables

**Table 1:** Studies included in the systematic review and meta-analysis.

Study	Study Group (SD)								Control Group								Country	Continent	Latitude
	Sample Dimension			Indices		Prev. Cavitation	Age (years)	Ratio M/F (%)	Sample Dimension			Indices		Prev. de Cavitation	Age (years)	Ratio M/F (%)			
	F	M	T	DMFT	dmft				F	M	T	DMFT	dmft						
[22]	15	38	53	N/A	1.84	30	11.09	253.3	N/A	N/A	N/A	N/A	N/A	N/A	N/A	N/A	Jordan	Asia	31.963
[23]	26	44	70	N/A	N/A	39	4.41	169.2	9	23	32	N/A	N/A	25	9.22	139.1	Israel	Asia	31.769
[24]	23	22	45	N/A	N/A	10	13	95.7	18	27	45	N/A	N/A	19	13	150	Portugal	Europe	41.150
[25]	36	60	96	2.45	4.44	90	10.15	166.7	N/A	N/A	N/A	N/A	N/A	N/A	N/A	N/A	Yemeni	Asia	15.369
[26]	N/A	N/A	45	0.71	0.31	10	12.7	N/A	N/A	N/A	45	1.42	0.42	19	12.8	N/A	Portugal	Europe	41.150
[20]	14	16	30	0.90	1	21	N/A	114.3	14	16	30	2.47	2.33	25	N/A	114.3	India	Asia	24.571
[27]	N/A	N/A	146	N/A	N/A	60	N/A	N/A	N/A	N/A	N/A	N/A	N/A	N/A	N/A	N/A	Colombia	South America	−10.300
[28]	26	35	61	N/A	N/A	26	9	134.6	38	45	83	N/A	N/A	39	9.43	118.4	Brasil	South America	−30.028
[21]	13	17	30	N/A	N/A	10	6.37	30.8	16	14	30	N/A	N/A	29	7.53	87.5	Brasil	South America	−21.764
[29]	36	25	61	0.36	1.84	23	9.15	69.4	32	20	52	0.49	0.98	34	9.80	62.5	Brasil	South America	−30.028
[30]	N/A	N/A	52	1.40	1	32	N/A	N/A	N/A	N/A	N/A	N/A	N/A	N/A	N/A	N/A	Saudi Arabia	Asia	21.427
[31]	43	76	119	N/A	N/A	81	14.19	176.7	N/A	N/A	N/A	N/A	N/A	N/A	N/A	N/A	Pakistan	Asia	24.926
[32]	67	77	144	N/A	N/A	52	N/A	114.9	N/A	N/A	N/A	N/A	N/A	N/A	N/A	N/A	Brasil	South America	−21.764
[33]	43	63	106	2.73	N/A	57	9.3	146.5	70	55	125	1.65	N/A	72	11.7	78.8	United Arab Emitades	Asia	25.277
[34]	N/A	N/A	35	1.54	0.60	24	N/A	N/A	N/A	N/A	N/A	N/A	N/A	N/A	N/A	N/A	India	Asia	28.645
[35]	N/A	N/A	9	N/A	N/A	4	9.11	N/A	N/A	N/A	8	N/A	N/A	3	9.75	N/A	Brasil	South America	−30.028
[36]	N/A	N/A	49	N/A	N/A	15	N/A	N/A	N/A	N/A	N/A	N/A	N/A	N/A	N/A	N/A	South Africa	Africa	−26.95

DMFT—Decayed, Missing, and Filled Teeth; dmft—decayed, missing, and filled primary teeth; F—female; M- Male; N/A—Not available; Prev.—prevalence; DS —Down syndrome; T—total.

## Appendix A

**Table A1:** PRISMA checklist.

Section and Topic	Item #	Checklist Item	Location Where Item Is Reported
**TITLE**			
Title	1	Identify the report as a systematic review.	1
**ABSTRACT**			
Abstract	2	See the PRISMA 2020 for Abstracts checklist.	1
**INTRODUCTION**			
Rationale	3	Describe the rationale for the review in the context of existing knowledge.	2
Objectives	4	Provide an explicit statement of the objective(s) or question(s) the review addresses.	2
**METHODS**			
Eligibility criteria	5	Specify the inclusion and exclusion criteria for the review and how studies were grouped for the syntheses.	3
Information sources	6	Specify all databases, registers, websites, organisations, reference lists and other sources searched or consulted to identify studies. Specify the date when each source was last searched or consulted.	3
Search strategy	7	Present the full search strategies for all databases, registers and websites, including any filters and limits used.	3
Selection process	8	Specify the methods used to decide whether a study met the inclusion criteria of the review, including how many reviewers screened each record and each report retrieved, whether they worked independently, and if applicable, details of automation tools used in the process.	3
Data collection process	9	Specify the methods used to collect data from reports, including how many reviewers collected data from each report, whether they worked independently, any processes for obtaining or confirming data from study investigators, and if applicable, details of automation tools used in the process.	3
Data items	10a	List and define all outcomes for which data were sought. Specify whether all results that were compatible with each outcome domain in each study were sought (e.g., for all measures, time points, analyses), and if not, the methods used to decide which results to collect.	3
	10b	List and define all other variables for which data were sought (e.g., participant and intervention characteristics, funding sources). Describe any assumptions made about any missing or unclear information.	4
Study risk of bias assessment	11	Specify the methods used to assess risk of bias in the included studies, including details of the tool(s) used, how many reviewers assessed each study and whether they worked independently, and if applicable, details of automation tools used in the process.	4
Effect measures	12	Specify for each outcome the effect measure(s) (e.g., risk ratio, mean difference) used in the synthesis or presentation of results.	3
Synthesis methods	13a	Describe the processes used to decide which studies were eligible for each synthesis (e.g., tabulating the study intervention characteristics and comparing against the planned groups for each synthesis (item #5)).	3,4
	13b	Describe any methods required to prepare the data for presentation or synthesis, such as handling of missing summary statistics, or data conversions.	3,4
	13c	Describe any methods used to tabulate or visually display results of individual studies and syntheses.	3,4
	13d	Describe any methods used to synthesize results and provide a rationale for the choice(s). If meta-analysis was performed, describe the model(s), method(s) to identify the presence and extent of statistical heterogeneity, and software package(s) used.	3,4
	13e	Describe any methods used to explore possible causes of heterogeneity among study results (e.g., subgroup analysis, meta-regression).	3,4
	13f	Describe any sensitivity analyses conducted to assess robustness of the synthesized results.	3,4
Reporting bias assessment	14	Describe any methods used to assess risk of bias due to missing results in a synthesis (arising from reporting biases).	4
Certainty assessment	15	Describe any methods used to assess certainty (or confidence) in the body of evidence for an outcome.	4
**RESULTS**			
Study selection	16a	Describe the results of the search and selection process, from the number of records identified in the search to the number of studies included in the review, ideally using a flow diagram.	4
	16b	Cite studies that might appear to meet the inclusion criteria, but which were excluded, and explain why they were excluded.	4–5
Study characteristics	17	Cite each included study and present its characteristics.	4–5
Risk of bias in studies	18	Present assessments of risk of bias for each included study.	7
Results of individual studies	19	For all outcomes, present, for each study: (a) summary statistics for each group (where appropriate) and (b) an effect estimates and its precision (e.g., confidence/credible interval), ideally using structured tables or plots.	6–13
Results of syntheses	20a	For each synthesis, briefly summarise the characteristics and risk of bias among contributing studies.	6–13
	20b	Present results of all statistical syntheses conducted. If meta-analysis was done, present for each the summary estimate and its precision (e.g., confidence/credible interval) and measures of statistical heterogeneity. If comparing groups, describe the direction of the effect.	6–13
	20c	Present results of all investigations of possible causes of heterogeneity among study results.	6–13
	20d	Present results of all sensitivity analyses conducted to assess the robustness of the synthesized results.	6–13
Reporting biases	21	Present assessments of risk of bias due to missing results (arising from reporting biases) for each synthesis assessed.	6–13
Certainty of evidence	22	Present assessments of certainty (or confidence) in the body of evidence for each outcome assessed.	6–13
**DISCUSSION**			
Discussion	23a	Provide a general interpretation of the results in the context of other evidence.	14–15
	23b	Discuss any limitations of the evidence included in the review.	14–15
	23c	Discuss any limitations of the review processes used.	14–15
	23d	Discuss implications of the results for practice, policy, and future research.	14–15
**OTHER INFORMATION**			
Registration and protocol	24a	Provide registration information for the review, including register name and registration number, or state that the review was not registered.	3
	24b	Indicate where the review protocol can be accessed, or state that a protocol was not prepared.	3
	24c	Describe and explain any amendments to information provided at registration or in the protocol.	3
Support	25	Describe sources of financial or non-financial support for the review, and the role of the funders or sponsors in the review.	-
Competing interests	26	Declare any competing interests of review authors.	-
Availability of data, code and other materials	27	Report which of the following are publicly available and where they can be found: template data collection forms; data extracted from included studies; data used for all analyses; analytic code; any other materials used in the review.	17–24

From: Page MJ, McKenzie JE, Bossuyt PM, Boutron I, Hoffmann TC, Mulrow CD, et al. The PRISMA 2020 statement: an updated guideline for reporting systematic reviews. BMJ 2021;372:n71. doi: 10.1136/bmj.n71. For more information, visit: http://www.prisma-statement.org/ (accessed on 1 January 2020).
